# OH in the Solar Spectrum

**DOI:** 10.6028/jres.063A.023

**Published:** 1959-12-01

**Authors:** Charlotte E. Moore, Herbert P. Broida

## Abstract

Revised identifications of OH lines in the solar spectrum have been made from the detailed laborarory analyses of the A ^2^Σ^+^−X^2^∏ bands. In the (0, 0), (1, 1), and (2, 2) bands a total of 175 solar lines are ascribed to OH unblended; 124 have OH as a partial contributor. Laboratory intensities along the branches of the separate bands have been used as a guide in making the solar identifications.

A new edition of solar spectrum wavelengths, identifications, and other relevant data, to replace the 1928 edition [1],^1^ is in the course of preparation. This program includes the revision of molecular as well as atomic identifications of solar lines. Separate papers giving the solar identifications of individual selected molecules are being prepared. The first paper, *CH in the Solar Spectrum*, has been published [2]. The present paper is the second of this series.

In 1928, 185 solar lines were attributed wholly or partially to OH. The identifications were based on early laboratory measurements of the λ3064 band by Grebe and Holtz [3], supplemented by Fowler’s work [4]. Subsequently, Shaw [5] extended the identifications.

In 1948 Dieke and Crosswhite [6] published an extensive analysis of nine bands of the A ^2^Σ^+^−X^2^∏ system of OH. They used an oxyacetylene flame as the light source, and photographed the spectrum from 2800 A to 3550 A in the second order of a 21-ft concave grating having 30,000 lines per inch, and set up in a Paschen mounting. For each line the intensity measurement is given. These measurements were carried out photographically. “The emulsion was calibrated with the help of iron lines of known intensity.”

This splendid analysis has been used for the present work. A preliminary report on the revised identifications of OH lines in the solar spectrum was given by the authors in 1957 [7, 8]. Since then some revisions have been made and the counts have been done in more detail. The results are summarized in table 1.

From a study of the intensity measurements along the individual branches it appears doubtful whether lines having a laboratory intensity of about 10 or less are present in the sun. Most of the accessible lines in the last G bands listed in table 1 are faint. High-dispersion solar spectra have the short-wave limit near 2950 A because of the ozone absorption in the earth’s atmosphere. Consequently, some of the OH bands cannot yet be examined in the solar spectrum over the entire range of laboratory observations. The (1, 0) and (2, 1) bands should be present, and may be detected when high-dispersion rocket spectra become available.

In tables 2, 3, and 4, the detailed analyses of the (0, 0), (1, 1), and (2, 2) bands, respectively, are given. These tables are arranged identically. Laboratory data are on the left, and solar data on the right. The rotational quantum numbers of the lines as assigned in the laboratory analyses are entered under the headings for the various branches, O, P, Q, R, and S. Primes denote satellite lines throughout. For example, in table 2, the line of wavelength 3066.114 A, is a member of the R_2 1_ branch, and has the quantum number 5 entered in the R_1_ column as 5′. Similarly, the line at 3090.270 A has quantum number 4 in the Q_1 2_ branch, entered as 4′ in the Q_2_ column.

Dieke and Crosswhite have assigned individual intensities in cases where a line is blended. If the blending occurs within the same band, the intensities are entered in the table in the order of the rotational lines as read from left to right. For example in table 2, the line at 3091.186 A is a blend. The intensity of the P_1_ contributor is 416; that of the Q_1 2_ satellite line is 83. In cases where the blending occurs in different bands, an asterisk follows the laboratory wave number.

The solar entries are from the current revision. The wavelengths are from the 1928 edition with a small running correction applied to convert them to the 1928 International Solar Standards [9]. The intensities are the eye estimates as given in Rowland’s table [10], with −3 substituted for Rowland’s 0000; −2 for 000; and −1 for 00, as was done in 1928.

The differences in wavelength “sun–lab.” indicate very good agreement between the measurements in the two sources. For example, in table 2, for the unblended lines the solar wavelengths average 0.007 A greater than the laboratory measurements. In general, residuals greater than ±0.030 A are tolerated only if the solar line is a blend or is extremely faint.

In assigning solar identifications, judgment enters into the picture inherently. Some investigators are more conservative than others. Complications arise from blending, i.e., a solar line may have more than one contributor to its chemical origin. Many solar lines are produced by blends of atomic and molecular lines. The present assignments are based on the best available data among laboratory spectra. The symbol “||” preceding the chemical symbol, denotes a predominant contributor and “|” indicates a contributor stronger than the others. A dash, “–”, in this column is used in the case of blends to distinguish the laboratory lines shorter than the solar wavelength from those that are longer. For example, in table 2, the laboratory wavelength of the Fe I line is less than the solar value 3078.044 A, while that of the OH line is greater. In the case of the solar line at 3082.035 A, OH is probably a contributor on the long-wave side of a solar line that is not yet completely accounted for as to chemical origin. If “OH” is entered in parentheses in this column, it is masked in the solar spectrum.

Under “Notes”, the four letters have the following meaning:

P OH present in the solar spectrum, unblended.
B OH present in the solar spectrum, blended.
M OH masked in the solar spectrum.
A OH absent from the solar spectrum.

For laboratory lines of OH that are masked by stronger unresolved components, the “OH” entry in the solar identification column applies to the stronger OH line, and the weaker ones are masked. For example see table 4, λ3226.443.

In table 4, only 58 of the total of 159 lines have been compared with the solar ledger. Lines of intensity fainter than about 10 are probably absent, and coincidences between laboratory and solar wavelengths are considered accidental for the fainter lines.

## Figures and Tables

**Table 1:** OH in the Solar Spectrum Summary

Laboratory					Sun					
Table No.	Electronic transition	Vibrational transition	Wavelength range A	Total number lines	Strongest solar int. Rowl. est.	Summary of counts				
						Present	Blend	Masked	Absent	Total
										
2	A ^2^Σ^+^−X^2^∏	0, 0	3021 to 3362	283	3	108	63	69	43	283
3		1, 1	3109 to 3378	231	1	52	56	81	42	231
4		2, 2	3184 to 3372	159	−1	15	5	23	15	58
										
			Total	673		175	124	173	100	572
										
		3, 3	3253 to 3356	73		0				
		1, 0	2811 to 3050	219		0				
		0, 1	3428 to 3545†	119						
		2, 1	2854 to 3070	186		0				
		1, 2	3483 to 3545†	56						
		3, 2	2944 to 3060	119		0				

† Wavelengths from one plate only; the authors state that the wave numbers should be increased by about 0.2 cm^−1^ (see ref. 6).

**Table 2:** OH in the Solar Spectrum A ^2^Σ+−X^2^∏ (0, 0)

Laboratory											Sun				
O_2_	P_1_	P_2_	Q_1_	Q_2_	R_1_	R_2_	S_1_	Intensity	Wave number cm^−1^	Wavelength A	Wavelength A	Disk int. Rowl. est.	⊙–lab. A	Solar identification	Notes

							15, 16	2+2+2	33088.89*	3021.285					A
							14, 17	3+1	33083.82	3021.749	3021 719	0	−0.030		M
							13	4	33073.92	3022.652					A
							18	1	33073.07	3022.730	3022.747	3	+0.017	Mn I	M
							12	5	33059.25	3023.994					A
							11	7	33039.96	3025.760					A
							10	9	33016.31	3027.927	3027.890	−1*N*	−0.037	−Pd I	M
							9	11	32988.40	3030.490	3030.484	−3	−0.006	OH?	P
							8	14	32956.39	3033.433	3033.434	4*d*?	+0.001	V II	M
							7	18	32920.61	3036.730	3036.754	2*N*	+0.024	−Ti II	M
							6	22+3	32881.22*	3040.368	3040.35	−3	−0.02	OH	P
							5	26	32838.48	3044.325	3044.333	−2	+0.008	OH	P
							4	29	32792.81	3048.565	3048.569	−3	+0.004	OH	P
							3	30	32744.63	3053.051	3053.068	3	+0.017	Fe I	M
							2	28	32694.55	3057.727					A
							1	19	32643.36	3062.523	3062.52	−1	0.00	OH?	P
					9			402	32632.25	3063.565	3063.555	3	−0.010	OH	P
					8, 10, 9′			415+378+44	32630.55	3063.725	3063.729	2	+0.004	OH	P
					8′			55	32628.46	3063.921	} 3063.936	2	$\{\begin{array}{c} +0.015\\ -0.034 \end{array}$	} Ni II—|| Fe I	$\{\begin{array}{c} M\\ M \end{array}$
					10′			31	32627.94	3063.970					
					7			415	32625.61	3064.189	} 3064.216	2	$\{\begin{array}{c} +0.027\\ -0.020 \end{array}$	OH–OH	$\{\begin{array}{c} B\\ B \end{array}$
					11			346	32625.11	3064.236					
					7′			68	32623.68	3064.370	3064.377	1	+0.007	Co I	M
					11′			27	32622.39	3064.491	3064.515	−2	+0.024	Nb II	M
					6			397	32617.51	3064.950	3064.955	1	+0.005	OH	P
					12, 6′			310+83	32615.97	3065.095	3065.094	2	−0.001	|OH Cr I	B
					12′			20	32613.02	3065.372					A
					5			363	32606.60	3065.976	3065.994	2	+0.018	OH–Mn I	B
					5′			100	32605.13	3066.114	3066.144	0	+0.030	OH–Al I	B
					13			271	32602.96	3066.318	3066.364	1	+0.046	Ti II V I	M
					13′			15	32599.82	3066.613					A
					4			304	32593.16	3067.240	3067.262	8	+0.022	Fe I	M
					4′			114	32591.92	3067.356	3067.386	1*N*	+0.030	OH–	B
						10		352	32588.68	3067.661	3067.657	1	−0.004	OH	P
						9		373	32587.47	3067.775	3067.781	1	+0.006	OH	P
					14	11		230+323	32585.84	3067.929	3067.939	2	+0.010	OH–Fe I	B
					14′	8		11+383	32582.14	3068.277	3068.281	1	+0.004	OH	P
						12		290	32578.63	3068.608	3068.598	1	−0.010	OH	P
					3			234	32577.61	3068.704	3068.725	1	+0.021	|OH–Fe II	B
					3′			126	32576.60	3068.799	3068.796	0	−0.003	OH	P
						7		378	32572.59	3069.177	3069.181	2	+0.004	OH	P
						13		255	32567.30	3069.675	3069.681	1	+0.006	OH V I	B
					15			193	32564.78	3069.913	3069.915	1	+0.002	OH	P
					15′			9	32561.27	3070.244	} 3070.265	3	$\{\begin{array}{c} +0.021\\ -0.053 \end{array}$	Mn I	$\{\begin{array}{c} M\\ M \end{array}$
					2			152	32560.48	3070.318					
					2′			127	32559.53	3070.392	3070.380	−1	−0.012	OH	P
						6		359	32558.79	3070.478	3070.492	1	+0.014	OH	P
						14		218	32551.72	3071.145	3071.145	1	0.000	OH–Fe II	B
					1			69	32542.56	3072.009	3071.965	1	−0.044	Co I	M
					1′			102	32541.99	3072.063	3072.115	3	+0.052	Ti II	M
						5		325	32540.55	3072.199	3072.182	0	−0.017	OH	P
					16			159	32539.38	3072.308	3072.328	3	+0.020	OH–|Co I	B
					16′			6	32535.67	3072.660	3072.670	−2	+0.010	OH?	P
						15		183	32531.76	3073.028	3072.984	6*Nd*?	−0.044	Ti II	M
						4		273	32517.58	3074.369	3074.385	1	+0.016	OH	P
					17			131	32509.61	3075.123	3075.135	0	+0.012	OH	P
						16		151	32507.38	3075.334	3075.355	0	+0.021	OH	P
					17′			5	32505.77	3075.486					A
						3		204	32489.49	3077.028	3077.027	0	−0.001	OH	P
						17		125	32478.48	3078.071	3078.044	4*d*?	−0.027	|Fe I–OH	B
					18			102	32475.28	3078.373	3078.387	2	+0.014	OH	P
			1					239	32474.58	3078.440	} 3078.445	3	$\{\begin{array}{c} +0.005\\ -0.023 \end{array}$	OH ||Fe I	$\{\begin{array}{r} B\\ M \end{array}$
			1′					166	32474.28	3078.468					
					18′			3	32471.28	3078.753	3078.662	8*d*?	−0.091	||Ti II Fe II	M
			2					437	32458.65	3079.951	} 3079.979	4	$\{\begin{array}{c} +0.028\\ -0.027 \end{array}$	|OH–Fe I	$\{\begin{array}{r} B\\ M \end{array}$
			2′					152	32458.07	3080.006					
						2		138	32455.70	3080.231	3080.245	0	+0.014	OH	P
						18		98	32444.91	3081.255	3081.247	2	−0.008	OH Fe I	B
			3					616	32441.90	3081.541	3081.550	1	+0.009	OH	P
			3′					130	32441.07	3081.620					A
	1							252	32440.60	3081.665	3081.680	1	+0.015	OH	P
					19			80	32436.38	3082.065	3082.035	1	−0.030	−OH	B
					19′			2	32432.27	3082.456					A
			4					766	32423.63	3083.278	3083.282	1	+0.004	OH	P
			4′					111	32422.62	3083.374	3083.382	−1	+0.008	OH	P
						1		68	32415.51	3084.050	3084.055	−1	+0.005	OH	P
						19		77	32406.64	3084.894	3084.897	1	+0.003	OH	P
			5					884	32403.47	3085.196	3085.206	2	+0.010	OH	P
			5′					93	32402.10	3085.317	3085.331	1	+0.014	Cr II	M
					20			66	32392.66	3086.226	3086.229	0	+0.003	OH	P
	2							335	32390.94	3086.390	3086.400	1	+0.010	Co I–OH	B
					20′			2	32388.38	3086.634	3086.636	−1	+0.002		M
			6					974	32380.99	3087.338	3087.345	1	+0.007	OH	P
			6′					77	32379.49	3087.481	3087.453	0	−0.028	Fe I–OH	B
						20		59	32363.49	3089.008	3089.000	0	−0.008	OH	P
			7					995	32355.88	3089.734	3089.745	2	+0.011	OH	P
			{ 7′	2,3 2′,3′				62+293+439+95+100	} 32354.55	3089.861	3089.868	2	+0.007	OH	P
				4′				94	32350.27	3090.270	3090.222	1*N*	−0.048	Fe I–Co I	M
				4				589	32349.29	3090.364	3090.374	1	+0.010	OH	P
				1′				70	32348.40	3090.449	} 3090.486	0	$\{\begin{array}{c} +0.037\\ +0.013 \end{array}$	OH OH	$\{\begin{array}{c} B\\ B \end{array}$
				1				139	32348.15	3090.473					
					21			47	32344.08	3090.862	3090.868	0	+0.006	OH	P
	3			5′				416+83	32340.69	3091.186	3091.213	1*N*	+0.027	CH ||OH	B
				5				712	32338.86	3091.361	3091.371	1	+0.010	OH	P
			8					1000	32328.06	3092.394	3092.403	1	+0.009	OH	P
			8′					50	32326.15	3092.577	3092.598	′3	+0.021	CH OH	B
				6′				71	32325.38	3092.650	3092.712	4	+0.062	Al I	M
				6				808	32323.96	3092.786	3092.851	1	+0.065	Al I (OH)	M
						21		45	32315.37	3093.609	3093.608	′1	′0.001	OH	P
		1′						143	32314.19	3093.722	3093.723	′2	+0.001	OH	P
				7′				58	32306.49	3094.459	3094.469	′1	+0.010	OH	P
				7				855	32304.83	3094.618	3094.626	2	+0.008	OH	P
			9					973	32297.38	3095.342	3095.347	3	+0.005	OH	P
			9′					40	32295.15	3095.546	3095.554	−1*N*	+0.008	OH	P
					22			35	32290.40	3096.000				OH Cr II	A
	4							492	32389.12	3096.124	3096.138	2	+0.014		B
		2, 2′						114+154	32286.76	3096.349	3096.324	0	−0.025	Fe II–OH	B
				8′				48	32283.62	3096.650	3096.624	0	−0.026	–OH	B
				8				873	32281.74	3096.830	3096.902	5	+0.072	Mg I	M
			10					912	32263.45	3098.586	3098.588	2	+0.002	OH	P
						22		34	32262.11	3098.715	3098.720	−2	+0.005	OH	P
			10′					31	32261.16	3098.807	3098.825	−1	+0.018	OH	P
				9′				38	32256.97	3099.210	3099.235	0	+0.025	Zr II OH	B
				9				860	32254.86	3099.411	3099.418	1	+0.007	OH	P
		3′						158	32253.54	3099.538	} 3099.575	0*N*	$\{\begin{array}{c} +0.037\\ -0.018 \end{array}$	OH–OH	$\{\begin{array}{c} B\\ B \end{array}$
2		3						33+215	32252.97	3099.593					
	5							546	32235.96	3101.229	3101.242	1	+0.013	OH	P
					23			26	32231.51	3101.657					A
			11	10′				842+30	32226.47	3102.142	3102.148	1	+0.006	OH	P
			11′	10				24+871	32224.23	3102.358	3102.369	2	+0.011	OH	P
		4′						148	32214.79	3103.267	3103.284	0	+0.017	OH	P
		4						307	32214.01	3103.342	3103.349	1	+0.007	OH	P
						23		25	32203.57	3104.348	3104.349	−1	+0.001	OH	P
				11′				23	32192.44	3105.421	3105.464	2	+0.043	Ni I	M
				11				763	32189.94	3105.663	3105.677	1	+0.014	OH	P
3			12					43+752	32186.27	3106.017	3106.032	2	+0.015	OH	P
			12′					18	32183.55	3106.279	3106.241	3	−0.038	Ti II	M
	6							582	32180.83	3106.542	3106.559	2	+0.017	Fe II |OH–Zr II	B
		5′						131	32171.36	3107.457	3107.459	0	+0.002	OH–Ti I	B
		5						384	32170.36	3107.553	3107.565	1	+0.012	|OH–Cr II	B
					24			19	32167.27	3107.852	3107.854	−1	+0.002		M
				12′				18	32154.66	3109.069	3109.073	1	+0.004	|Fe I–Hf II	M
				12				690	32151.97	3109.330	3109.333	3	+0.003	OH CH	B
			13					664	32142.73	3110.223	3110.245	5*Nd?*	+0.022	OH–	B
			13′			24		14+18	32139.70	3110.517	3110.529	−1	+0.012	OH	P
	7	6′						607+111	32123.54	3112.082	3112.077	2	−0.005	Ti II–|Fe I |OH	B
		6						441	32122.52	3112.181	3112.214	1*N*	+0.033	OH	P
4				13′				44+14	32113.29	3113.075	3113.097	−1	+0.022	OH	P
				13				611	32110.35	3113.361	3113.384	1	+0.023	OH	P
					25			14	32097.35	3114.622	3114.628	−1	+0.006		M
			14					569	32095.83	3114.769	3114.778	1	+0.009	OH	P
			14′					11	32092.65	3115.077	3115.043	1*N*	−0.034	Fe I	M
		7′						94	32072.36	3117.048	3117.037	−1	−0.011	OH	P
		7						481	32070.89	3117.191	3117.201	1	+0.010	OH	P
						25		13	32070.03	3117.275	3117.249	0	−0.026	Cr II	M
				14′				10	32068.18	3117.455	3117.432	−1*N*	−0.023	Ti I	M
				14				527	32065.05	3117.759	3117.768	1	+0.009	OH	P
	8							609	32063.74	3117.886	3117.890	2	+0.004	OH–Ti I	B
			15					478	32045.43	3119.668	3119.678	1	+0.010	OH CH–Cr I	B
			15′					8	32042.10	3119.992	3120.012	−1*N*	+0.020	OH–Fe II	B
5								41	32036.08	3120.578	3120.602	−2	+0.024	OH	P
					26			10+11	32021.98*	3121.953	3121.969	−1	+0.016	Cr II	M
				15′				8+63	32019.34*	3122.210	3122.219	0	+0.009	OH CH	M
		8′						76	32017.43	3122.397					A
				15				447	32016.08	3122.528	} 3122.570	2	$\{\begin{array}{c} +0.042\\ +0.004 \end{array}$	} OH OH–Cr II	B
		8						493	32015.69	3122.566					B
	9							531+48	32001.56*	3123.945	3123.959	1	+0.014	OH	P
						26		10	31994.69	3124.616	3124.638	−1	+0.022	OH?	P
			16					399	31991.48	3124.929	3124.918	2	−0.011	CH OH	B
			16′					6	31988.11	3125.258	3125.288	5	+0.030	V II	M
				16′				6+53	31966.74*	3127.347	3127.362	0	+0.015	OH	M
				16				372	31963.32	3127.682	3127.671	2*d*?	−0.011	|OH CH	B
		9′						61	31959.14	3128.091	3128.086	0*N*	−0.005	OH—OH	B
		9						492+16	31957.20*	3128.281	3128.289	1	+0.008	Sc II |OH	B
6								36+48	31954.81*	3128.515	3128.521	0	+0.006	OH	P
					27			7	31940.34	3129.933	3129.947	−1*N*	+0.014	Y II?	M
	10							548	31936.84	3130.276	3130.267	3	−0.009	|V II–OH	B
			17					324	31933.87	3130.567	3130.567	1	0.000	|OH Fe II	B
			17′					4	31930.19	3130.928					A
						27		7	31913.37	3132.578					A
				17′				4	31910.47	3132.863					A
				17				305	31906.78	3133.225	3133.216	1	−0.009	OH	P
		10′						49	31897.55	3134.132	3134.116	8	−0.016	Fe I |Ni I	M
		10						475	31895.44	3134.339	3134.337	1	−0.002	|OH Cr II	B
			18					265	31872.57	3136.588	3136.590	0	+0.002	OH	P
7	11							31+505	31869.52	3136.888	3136.890	1	+0.002	OH	P
			18′					3	31868.61	3136.978					A
					28			5	31852.68	3138.547	3138.518	2	−0.029	Fe I	M
				18′				3	31850.29	3138.783	3138.786	0	+0.003		M
				18				250	31846.40	3139.166	3139.164	2	−0.002	OH	P
		11′						38	31832.84	3140.503	3140.511	−2	+0.008	OH	P
		11						445+9	31830.53*	3140.731	3140.757	3	+0.026	|OH–Ca I	B
						28		5+22	31825.88*	3141.190	3141.181	0	−0.009	Ca I	M
			19					205	31807.44	3143.011	3143.016	1	+0.005	OH	P
			19′					2	31803.39	3143.412					A
	12							459	31799.49	3143.797	3143.764	4	−0.033	|Ti II CH–OH	B
				19′				2	31786.16	3145.115	3145.091	3	−0.024	|Fe I–Cr II	M
				19				194	31782.09	3145.518	} 3145.526	{ 0	+0.008	OH	P
8								26	31781.47	3145.579			−0.053		M
		12′						30+15	31765.06*	3147.195	3147.235	3	+0.040	Cr II	M
		12						407	31762.53	3147.456	3147.447	1	−0.009	OH–|OH	B
					29			3	31758.54	3147.851					A
			20					159	31738.44	3149.844	3149.852	2	+0.008	OH	P
						29		3	31731.99	3150.485	3150.512	−2	+0.027		M
	13							405	31726.79	3151.001	3151.005	1	+0.004	OH	P
				20				151	31713.78	3152.293	3152.262	5	−0.031	||Ti II–OH	B
		13′						23	31694.26	3154.235	3154.200	3	−0.035	|Fe II Ti II	M
		13						363	31691.53	3154.507	3154.493	1	−0.014	OH Fe I	B
9								21+143	31690.39*	3154.621	3154.643	0	+0.022	OH	P
			21					122	31665.38	3157.112	3157.143	1	+0.031	|OH–Fe IP	B
					30			2	31657.83	3157.865	3157.882	1	+0.017	||Fe I–V II	M
	14							350	31651.40	3158.507	3158.521	0	+0.014	OH	P
				21				115	31641.40	3159.505	3159.531	1	+0.026	|Ni I OH–Cr I	B
						30		2	31631.34	3160.510					A
		14′						17	31620.45	3161.598					A
		14						318	31617.51	3161.892	3161.901	0	+0.009	OH	P
10								17	31595.81	3164.064	3164.068	0	+0.004	CH	M
			22					92	31588.27	3164.819	3164.833	1	+0.014	|V II OH	B
	15							297	31573.14	3166.336	3166.335	0	−0.001	OH	P
				22				88	31564.85	3167.168	3167.177	1	+0.009	OH	P
					31			1	31550.49	3168.609					A
		15′						13	31543.65	3169.296					A
		15						272+7	31540.50*	3169.613	3169.616	0	+0.003	OH	P
						31		1	31523.80	3171.292					A
			23					68	31506.92	3172.991	3172.997	1	+0.006	OH	P
11								14	31498.73	3173.816	3173.840	−2	+0.024	OH	P
	16							248	31492.12	3174.482	3174.490	1	+0.008	OH	P
				23				65+2	31484.02*	3175.299	3175.314	1	+0.015	OH Fe I	B
		16′						10	31463.81	3177.339	3177.302	2	−0.037	Co I–CH	M
		16						228	31460.48	3177.675	3177.680	−1	+0.005	OH	P
			24					50	31421.22	3181.645	3181.641	−1	−0.004	OH	P
	17							186	31408.19	3182.965	3182.990	3	+0.025	||Fe I–Ni I	M
						32		1+1	31406.08	3183.180					A
12				24				11+48	31398.78	3183.919	3183.964	2	+0.045	V I	M
		17′						7	31380.97	3185.727					A
		17						188+3+3	31377.45*	3186.084	3186.104	0*Nd?*	+0.020	OH	P
			25					37	31331.06	3190.802	3190.849	2	+0.047	Fe I	M
	18							164	31321.42	3191.784	3191.799	−1	+0.015	OH	P
				25				35	31308.97	3193.053	3193.054	−1	+0.001	OH	P
13								8	31296.74	3194.301					A
		18′						5	31295.11	3194.467					A
		18						158	31291.38	3194.848	3194.849	0	+0.001	|OH Ce II?	B
			26					26	31236.22	3200.489	3200.469	5*Nd?*	−0.020	Ni I–||Fe I	M
	19							133	31231.62	3200.961	3200.962	1	+0.001	OH	P
				26				25	31214.52	3202.715	3202.695	0	−0.020	CH OH	B
		19′						4	31206.13	3203.576					A
		19						124+2	31202.23*	3203.977	3203.980	1	+0.003	OH	P
14								6	31191.94	3205.034					A
	20							102	31138.83	3210.500	3210.480	1	−0.020	OH	P
			27					19	31136.65	3210.725	3210.724	−1	−0.001	CH OH	B
				27				18	31115.28	3212.934	3212.892	2*N*	−0.042	Mn I	M
		20′						3	31113.99	3213.063					A
		20						95+4	31109.97*	3213.479	3213.474	−1	−0.005	OH	P
15								5	31084.69	3216.092					A
	21							78	31042.95	3220.420	3220.433	0	+0.013	OH	P
			28					13	31032.05	3221.548	3221.545	−2	−0.003	OH	P
		21′						2	31018.77	3222.927	3222.944	−2	+0.017	Fe II?	M
		21						74	31014.52	3223.369	3223.364	−1	−0.005	OH	P
				28				13	31011.01	3223.734	3223.744	−2	+0.010	OH?	P
16								4	30975.13	3227.468					A
	22							60	30943.87	3230.729	3230.727	1	−0.002	OH |Mn I	B
			29					9	30922.38	3232.974	3232.938	2	−0.036	Ni I	M
		22′						1	30920.28	3233.193	3233.167	−1	−0.026	Ni I	M
		22						56	30915.87	3233.654	3233.669	−1	+0.015	OH	P
				29				9	30901.59	3235.149					A
17								3+1	30863.24*	3239.170					A
	23							45	30841.55	3241.447	3241.489	0	+0.042	Fe I	M
		23						42+35	30813.99*	3244.346	3244.354	0*N*	+0.008	OH	P
			30					6	30807.15	3245.067					A
				30				6	30786.72	3247.220					A
18								2	30748.87	3251.218					A
	24							33	30735.83	3252.597	3252.609	0*N*	+0.012	OH	P
		24						32	30708.50	3255.491	3255.497	1	+0.006	OH–	B
			31					4	30686.32	3257.812	3257.823	1	+0.011	Cr I	M
				31				4	30666.26	3259.976	3259.989	4	+0.013	||Fe I Cr I	M
19								1	30632.23	3263.597					A
	25							24	30626.72	3264.185	3264.185	−1*N*	0.000	OH	P
		25						23	30599.58	3267.080	3267.062	1	−0.018	Fe II–OH	B
				32				3	30539.85	3273.740	3273.720	−3	−0.020		M
	26							17	30514.00	3276.243	3276.262	−1	+0.019	OH	P
		26						17	30487.11	3279.133	3279.154	1	+0.021	OH	P
	27							12	30397.55	3288.795	3288.813	0	+0.018	Zr II	M
		27						12	30370.91	3291.679	3291.697	−1	+0.018	Fe I	M
	28							9	30277.26	3301.862	3301.869	−3	+0.007	OH? Pt I	B
	28							8	30250.81	3304.749	3304.754	−1*N*	+0.005	OH?–	B
29								6	30153.06	3315.462					A
	29							6	30126.65	3318.369	3318.367	1*Nd?*	−0.002	Ti I–Co I	M
30								4	30024.67	3329.641	3329.632	−3*N*	−0.009	Fe I	M
	30							4	29998.38	3332.558	3332.576	−3*N*	+0.018		M
31								3	29891.82	3344.439					A
	31							3	29865.33	3347.371	3347.375	−2	+0.004		M
32								2	29754.34	3359.892					A
	32							2	29728.27	3362.839					A

* Blend.

**Table 3:** OH in the Solar Spectrum A ^2^Σ^+^−X^2^∏ (1, 1)

Laboratory											Sun				
O_2_	P_1_	P_2_	Q_1_	Q_2_	R_1_	R_2_	S_1_	Intensity	Wave number cm^−1^	Wavelength A	Wavelength A	Disk int. Rowl. est.	⊙–lab. A	Solar identification	Note:

							3	5	32147.10	3109.801	3109.803	−3	+0.002	OH?	P
							2	4	32101.06	3114.262					A
							1	3	32053.54	3118.879					A
					8			67	32025.16	3121.643	3121.604	3	−0.039	Ti II ‖Co I	M
					7			68	32023.93	3121.762	3121.783	1	+0.021	OH |Fe I	B
					9,8′			65+9	32022.86	3121.867	3121.859	−1	−0.008	Cr II–OH	B
					7′			11+10	32021.98*	3121.953	3121.969	−1	+0.016	Cr II	M
					9′			7	32020.56	3122.091	3122.079	0	−0.012	Ti II	M
					6			63+8	32019.34*	3122.210	3122.219	0	**+**0.009	OH CH	B
					6′			13	32017.81	3122.360					A
					10			62	32016.73	3122.465					A
					10′			6	32014.36	3122.696	3122.664	−1	−0.032	Fe I	M
					5			57	32011.67	3122.958	3122.949	−1	−0.009	OH	P
					5′			16	32010.25	3123.096	3123.092	0	−0.004	Ti I	M
					11			57	32006.79	3123.434	3123.443	−1	**+**0.009	OH	P
					11′			4	32004.20	3123.687	3123.698	−3	**+**0.011	Fe II?	M
					4			48+531	32001.56*	3123.945	3123.959	1	**+**0.014	OH	M
					4′			18	32000.03	3124.094	3124.097	0	**+**0.003	Fe I	M
					12			51	31992.79	3124.801	3124.803	−1	**+**0.002	OH–Ge I	B
					12′			3	31990.07	3125.067	3125.053	2	−0.014	Cr II–CH	M
					3			37	31988.34	3125.236	} 3125.288	5	$\{\begin{array}{c} +0.052\\ -0.041 \end{array}$	} V II	$\{\begin{array}{c} M\\ M \end{array}$
					3′			20	31987.38	3125.329					
						9		60	31977.16	3126.329	3126.332	−2	**+**0.003	OH	P
						8		61	31975.78	3126.464	3126.472	−1	**+**0.008	OH	P
					13			45	31974.59	3126.580	} 3126.617	1	$\{\begin{array}{c} +0.037\\ -0.001 \end{array}$	CH OH OH	$\{\begin{array}{c} B\\ B \end{array}$
						10		57	31974.20	3126.618					
					2			24	31973.63	3126.674					A
					2′			20	31972.91	3126.745	3126.767	1	**+**0.022	Fe I	M
					13′			3	31971.62	3126.871	3126.847	0	−0.024	Fe IP	M
						7		62	31969.91	3127.038	3127.047	−1	**+**0.009	OH	P
						11		53+6	31966.74*	3127.347	3127.362	0	**+**0.015	OH	P
						6		57	31959.51	3128.055	3128.086	0*N*	+0.031	OH–OH	B
					1			11	31957.78	3128.224					A
					1′			16+492	31957.20*	3128.281	3128.289	1	+0.008	Sc II |OH	M
						12		48+36	31954.81*	3128.515	3128.521	0	+0.006	OH	P
					14			39	31952.12	3128.779	3128.776	−1	−0.003	OH Y II?	B
					14′			2	31948.91	3129.093	3129.107	1	+0.014	Fe I	M
						5		51	31944.43	3129.532	3129.532	−1*N*	0.000	OH	P
						13		43	31938.40	3130.123	3130.137	−1*N*	+0.014	|OH–Ti I	B
					15			33	31925.20	3131.418	3131.446	0	+0.028	OH	P
						4		43	31924.40	3131.496	3131.526	0	+0.030	OH–Cr II	B
					15′			2	31921.78	3131.753					A
						14		37	31917.40	3132.182	3132.189	0	+0.007	OH	P
						3		32	31899.00	3133.989	3133.966	−1*d?*	−0.023	Fe IP–OH	B
					16			28	31893.67	3134.514	} 3134.541	−2	$\{\begin{array}{c} +0.027\\ -0.037 \end{array}$	} OH–OH	$\{\begin{array}{c} B\\ B \end{array}$
			1					37	31893.01	3134.578					
			1′					26	31892.80	3134.599	3134.626	−1	+0.027	OH	P
						15		31	31891.72	3134.705	3134.716	−1	+0.011	OH Hf II	B
					16′			1	31890.01	3134.873					A
			2					68	31876.78	3136.174	} 3136.195	0	$\{\begin{array}{c} +0.021\\ -0.032 \end{array}$	OH |Fe I	$\{\begin{array}{c} B\\ M \end{array}$
			2′					24	31876.24	3136.227					
						2		22	31867.82	3137.056	3137.025	0	−0.031	Co I?- OH	B
						16		26	31861.28	3137.705	3137.710	−1	+0.005	OH	P
	1							39	31860.78	3137.749	3137.765	0	+0.016	Co I–OH	B
			3					96	31859.31	3137.894	3137.896	−1	+0.002	OH	P
			3′					20	31858.55	3137.969					A
					17			23	31857.39	3138.083	3138.076	−1	−0.007	V II–OH	B
					17′			1	31853.65	3138.452					A
			4					120	31840.07	3139.791	3139.761	1	−0.030	||V II Sc II–OH	B
			4′					17	31839.10	3139.886					A
						1		9+445	31830.53*	3140.731	3140.757	3	+0.026	|OH–Ca I	M
						17		22+5	31825.88*	3141.190	3141.181	0	−0.009	Ca I	M
			5					139	31818.60	3141.909	3141.908	2	−0.001	–OH	B
			5′					15	31817.44	3142.023	3142.021	−2	−0.002	OH	P
					18			18	31816.17	3142.142	3142.156	−2	+0.014	OH–V II?	B
	2							52	31812.39	3142.522	3142.511	−2	−0.011	OH	P
			6					152	31794.56	3144.284	3144.326	0	+0.042	CH |OH?	B
			6′					12	31793.15	3144.424	3144.453	1	+0.029	CH Cr I	M
						18		17	31785.59	3145.172	3145.136	2	−0.036	Ni I–	M
				2′				15	31772.13	3146.504					A
				2, 3′				43+16	31771.57	3146.560	} 3146.598	1	$\{\begin{array}{c} +0.038\\ -0.033 \end{array}$	} CH–CH OH	$\{\begin{array}{c} M\\ B \end{array}$
				3				68	31770.85	3146.631					
					19			15	31770.03	3146.712					A
			7					158	31767.67	3146.946	3146.934	1	−0.012	OH	P
			7′	1,1′				10+22+11	31766.00	3147.112					A
				4′				15+30	31765.06*	3147.195	3147.235	3	+0.040	Cr II	M
				4				93	31764.36	3147.274	3147.267	2	−0.007	Fe I–OH	B
	3							65	31762.99	3147.410	3147.447	1	+0.037	OH–|OH	B
				5′				13	31753.91	3148.309	3148.307	−1	−0.002	–OH?	B
				5				112	31752.72	3148.427	3148.440	3	+0.013	|Fe I OH Cr I	B
						19		14	31740.03	3149.687					A
			8	6′				163+11	31737.73	3149.915	3149.898	1	−0.017	OH	P
			8′	6				8+127	31736.31	3150.056	3150.077	1	+0.021	OH–Cr II	B
		1′						22	31733.71	3150.314	3150.307	1	−0.007	–OH	B
					20			12	31718.69	3151.806					A
				7′				9	31717.10	3151.964					A
				7				136	31715.47	3152.125	3152.117	0	−0.008	OH	P
	4							76	31712.16	3152.454	3152.457	−1	+0.003	OH	P
		2′						24	31707.21	3152.947	} 3152.957	0	$\{\begin{array}{c} +0.010\\ -0.010 \end{array}$	} OH–OH	$\{\begin{array}{c} B\\ B \end{array}$
		2						18	31707.01	3152.967					
			9					156	31704.62	3153.204	3153.191	3	−0.013	|Fe I OH	B
			9′					6	31702.60	3153.405					A
				8′				8	31692.20	3154.445	3154.420	1	−0.025	Fe I	M
				8				143+21	31690.39*	3154.621	3154.595	0	−0.026	Ni I–|OH	B
						20		11	31689.32	3154.727					A
		3′						25	31674.69	3156.184	3156.190	−3	+0.006	OH	P
		3						33	31674.12	3156.241	3156.272	2	+0.031	Fe I	M
			10					148	31668.20	3156.831	3156.845	0	+0.014	OH	P
			10′					5	31665.98	3157.052	3157.031	2	−0.021	Fe I	M
				9′				6	31663.32	3157.317	3157.294	0	−0.023		M
					21			9	31661.95	3157.454					A
				9				138	31661.26	3157.523	3157.501	1N	−0.022	–OH	B
	5							85	31659.36	3157.712	3157.751	0	+0.039	CH OH?	B
		4′						23	31636.55	3159.989					A
		4						48	31635.77	3160.067	3160.082	−1	+0.015	OH–Cr II	B
						21		8	31633.08	3160.336	3160.347	1	+0.011	Fe I	M
				10′				5	31630.38	3160.605	3160.612	−1	+0.007	Cr I CH	M
			11	10				137+133	31628.22	3160.821	3160.801	1	−0.020	V II–|OH	B
			11′					4	31625.95	3161.048	3161.033	0	−0.015	Mn I	M
3								7	31610.35	3162.609	3162.570	4	−0.039	Ti II	M
	6							92	31604.30	3163.214	3163.223	0	+0.009	OH	P
					22			7	31599.67	3163.678	3163.683	−3	+0.005		M
		5′		11′				20+4	31593.48	3164.297	3164.295	1	−0.002	Fe I Zr II	B
		5						60	31592.51	3164.394	3164.418	0	+0.024	OH	P
				11				124	31590.97	3164.548	3164.548	0	0.000	OH	P
			12					124	31584.96	3165.151	3165.157	1	+0.006	OH Fe IP	B
			12′					3	31582.40	3165.407	3165.420	−1	+0.013	Zr II	M
						22		6	31571.17	3166.534					A
				12′				3	31552.55	3168.402					A
				12				114	31549.90	3168.668	3168.672	0	+0.004	OH	P
	7							95	31546.77	3168.982	3168.955	1	−0.027	–OH	B
		6′						18	31546.02	3169.058	3169.075	−2	+0.017	OH–Fe IP	B
		6						69	31544.86	3169.174	3169.192	−2	+0.018	|OH–Cr II	B
4								7+272	31540.50*	3169.613	3169.616	0	+0.003	OH	M
			13					110	31537.98	3169.866	3169.861	0	−0.005	OH	P
			13′					2	31535.18	3170.148	3170.128	−1	−0.020		M
					23			5	31531.66	3170.502	3170.481	−1	−0.021		M
				13′				2	31508.67	3172.815					A
				13				101	31504.86	3173.198	3173.210	0	+0.012	OH	P
						23		5	31503.56	3173.329					A
		7′						15	31494.57	3174.235	3174.221	0	−0.014	Fe IP	M
		7						75	31493.16	3174.377	3174.380	0	+0.003	OH	P
			14					96	31487.30	3174.968	3174.953	1	−0.015	OH	P
	8							95	31486.60	3175.039	3175.045	0	+0.006	|Sn I OH	B
			14′					2+65	31484.02*	3175.299	3175.314	1	+0.015	OH Fe I	M
5								6	31464.92	3177.227					A
				14′				2	31458.98	3177.826	3177.822	−1*d*?	−0.004		M
					24			4	31457.66	3177.960					A
				14				89	31455.88	3178.140	3178.161	1	+0.021	OH	P
		8′						12	31439.22	3179.824					A
		8						78	31437.64	3179.984	3179.966	−1	−0.018	OH	P
			15					82	31432.83	3180.470	3180.491	0	+0.021	OH	P
			15′			24		1+4	31429.95	3180.762	3180.746	2	−0.016	Fe I	M
	9							95	31423.71	3181.393	3181.420	0	+0.027	OH Cr II	B
				15′				1+1	31406.08*	3183.180					A
				15				76	31402.83	3183.509	3183.520	−2	+0.011	OH	P
6								6	31385.40	3185.277					A
		9′						10	31380.30	3185.795	3185.804	−2*N*	+0.009	OH	P
		9						81	31378.50	3185.977	3185.979	0	+0.002	OH	P
					25			3+188+3	31377.45*	3186.084	3186.104	0*Nd*?	+0.020	OH	M
			16					69	31374.47	3186.387	3186.383	0	−0.004	OH	P
			16′					1+9	31370.78*	3186.762	3186.752	3	−0.010	Fe II	M
	10							88	31358.00	3188.010	3188.034	1	+0.024	OH Cr I	B
				16′		25		1+3	31350.14	3188.860					A
				16				64	31345.70	3189.312	3189.317	0	+0.005	OH	P
		10′						8+8	31317.74*	3192.158					A
		10						76	31315.80	3192.356	3192.396	0	+0.040	Fe I	M
			17					57+7	31312.12*	3192.732	3192.724	−2	−0.008	OH	P
			17′					1	31308.07	3193.145					A
7								5	31302.07	3193.757	3193.734	−1*N*	−0.023	Fe IP Fe IIP	M
	11							82	31289.40	3195.051	3195.085	0	+0.034	CH OH	B
				17′				1	31288.59	3195.133	3195.140	−1	+0.007	Ru II CH	M
				17				54	31284.47	3195.554	3195.593	2	+0.039	Ni I–Y II	M
						26		2, 3	31264.35*	3197.610	3197.596	−1	−0.014	V II?–CH	M
		11′						6	31251.95	3198.879	3198.902	−2*N*	+0.023	Fe I?–Ir I?	M
		11						72+3	31249.76*	3199.103	3199.137	0	+0.034	OH–	B
			18					46+3	31245.66*	3199.523	3199.527	4	+0.004	Fe I	M
				18				43	31219.00	3202.255	3202.257	−1	+0.002	OH	P
	12							75	31217.83	3202.376	3202.382	0	+0.006	|V I OH	B
8								4	31215.09	3202.657	3202.667	0	+0.010	Fe IP	M
		12′						5	31182.67	3205.986	3206.007	1	+0.021	Ti II	M
		12						67	31180.23	3206.237	3206.238	0	+0.001	OH	P
			19					37	31175.02	3206.773	3206.763	0*Nd*?	−0.010	–OH	B
				19				35+14	31149.18*	3209.433	3209.434	−1	+0.001	OH	P
	13							67	31143.17	3210.043	3210.046	0	+0.003	OH	P
9								3+3	31124.70*	3211.958					A
		13′						4+95	31109.97*	3213.479	3213.474	−1	−0.005	OH	M
		13						60	31107.44	3213.740	3213.744	0	+0.004	OH–Fe I	B
			20					29	31100.10	3214.499	3214.494	−2	−0.005	OH	P
				20				27	31075.04	3217.091	3217.097	1	+0.006	V I |V II	M
	14							58+5+4	31065.67*	3218.061	3218.075	−1	+0.014	OH	P
		14′						3	31034.10	3221.335					A
10		14						3+53	31031.29	3221.627	3221.659	2	+0.032	Ni I	M
			21					22	31020.73	3222.750	3222.729	−2*N*	−0.021	Ti I	M
				21				21	30996.33	3225.260	3225.267	−1	+0.007	OH	P
	15							50+9+3	30984.97*	3226.443	3226.446	−1	+0.003	OH	P
		15′						2	30954.87	3228.579					A
		15						46	30951.88	3228.892	3228.900	0	+0.008	|Fe I OH	B
			22					17+10+1	30936.75*	3231.473	3231.472	−1	−0.001	OH	P
11								2	30934.68	3231.689	3231.707	1	+0.018	Fe II Zr II	M
				22				16	30913.01	3233.954	3233.976	2	+0.022	||Fe I Mn I	M
	16							42	30901.10	3235.200	3235.187	−1	−0.013	Fe I–OH	B
		16′						2	30872.32	3238.216	3238.213	−2	−0.003	Ti I	M
		16						39	30869.17	3238.547	3238.553	−1	+0.006	OH	P
			23					13	30848.20	3240.748					A
12								2	30835.17	3242.118	3242.108	−3*N*	−0.010		M
				23				13	30824.92	3243.196	3243.214	−2	+0.018	OH	P
	17							35+42	30813.99*	3244.346	3244.354	0*N*	+0.008	OH	P
		17						32	30782.97	3247.615	3247.569	10	−0.046	Cu I	M
			24					9+12	30754.71*	3250.600	3250.637	3*N*	+0.037	Fe I	M
				24				9	30731.86	3253.017	3253.038	−2	+0.021	OH	P
	18							29+2+2	30723.76*	3253.875	3253.844	2*N*	−0.031	Fe I	M
		18						27	30693.41	3257.092	3257.103	2	+0.011	OH–|Fe I	B
			25					7	30656.06	3261.061	3261.065	−1	+0.004	Cd I	M
				25				7+1+1	30633.70*	3263.441	3263.466	−3	+0.025	Fe IP	M
	19							23	30630.09	3263.826	3263.838	0*N*	+0.012	OH–	B
		19						22	30600.42	3266.990	3266.950	1	−0.040	Fe II	M
			26					5	30552.13	3272.154					A
	20							18	30532.97	3274.208	3274.226	1	+0.018	OH–||Fe IP	B
				26				5	30530.13	3274.513					A
		20						17+7	30503.90*	3277.328	3277.358	7*d*?	+0.030	Fe II	M
			27					3	30442.80	3283.906	3283.933	−1	+0.027		M
	21							14	30432.37	3285.032	3285.022	0*N*	−0.010	|V II OH	B
				27				3+1	30421.17*	3286.242	3286.258	−2	+0.016	Sm II	M
		21						14	30403.78	3288.121	3288.155	3	+0.034	Ti II–	M
	22							11	30328.14	3296.322					A
			28					2+5	30327.68*	3296.372	3296.377	−2*N*	+0.005	Zr II	M
				28				2	30306.32	3298.695	3298.691	2	−0.004	Co I	M
		22						10	30299.95	3299.389					A
	23							8+4	30220.11*	3308.106	3308.111	0*N*	+0.005	NH	M
		23						8	30192.40	3311.142	3311.110	0	−0.032	NH	M
	24							6	30108.36	3320.385	3320.379	−3	−0.006		M
		24						6	30081.91	3323.415	3323.395	1	−0.020		M
	25							5	29992.50	3333.212	3333.222	−2*N*	+0.010		M
		25						5	29965.35	3336.232	3336.260	2	+0.028	Fe I	M
	26							4	29872.41	3346.611	3346.602	−3*N*	−0.009		M
		26						4	29845.53	3349.627	3349.652	−1	+0.025	Cr II?	M
	27							3	29748.07	3360.601	3360.607	0	+0.006	NH	M
		27						3	29721.36	3363.621	3363.616	1	−0.005	Ni I	M
	28							2	29619.12	3375.232	3375.215	−3	−0.017	Co I?	M
		28						2	29592.56	3378.261					A

* Blend.

**Table 4:** OH in the Solar Spectrum A ^2^Σ^+^−X^2^∏ (2, 2)

Laboratory										Sun				
O_2_	P_1_	P_2_	Q_1_	Q_2_	R_1_	R_2_	Intensity	Wave number cm^−1^	Wavelength A	Wavelength A	Disk int. Rowl. est.	⊙–lab. A	Solar identification	Notes

					7		10	31391.16	3184.692					A
					6		9	31390.37	3184.772					
					7′		2	31389.41	3184.869					
					6′		2	31388.82	3184.930					
					8		10	31388.31	3184.981					A
					5, 8′		8+1	31386.21	3185.195					
					5′		2	31384.81	3185.337					
					9		10	31381.60	3185.663	3185.674	0	+0.011		M
					9′		1	31379.49	3185.877					
					4		7	31379.00	3185.927					
					4′		3+188+3	31377.45*	3186.084					
					10		9+1	31370.78*	3186.762	3186.752	3	−0.010	Fe II	M
					3, 10′		5+1	31368.88	3186.955					
					3′		3	31367.97	3187.047					
					2		4	31356.63	3188.200					
					11, 2′		9+3	31355.76	3188.289					A
					1, 1′		2+3	31342.84	3189.604					
						8	9	31337.94	3190.101	3190.104	1	+0.003	CH	M
					12	7	8+9	31336.18	3190.280	3190.294	−2	+0.014	OH	P
						9	9	31335.04	3190.397	3190.404	−2	+0.007	OH?	P
						6	9	31329.35	3190.976					
						10	9	31327.37	3191.178					
						5	8+8	31317.74*	3192.158					
						11	8	31314.95	3192.443					
					13		7+57	31312.12*	3192.732					
						4	6	31300.75	3193.892					
						12	8	31297.66	3194.207					
					14		6	31283.65	3195.638					
			1, 1′				5+4	31281.46	3195.862					
						3	5	31278.35	3196.189					
						13	7	31275.35	3196.485					
			2				10	31264.87	3197.557	3197.541	1	−0.016	Ti II Fe I	M
			2′				3+2	31264.35*	3197.610					
	1						6	31250.96	3198.980					
					15		5	31250.13	3199.065					
						2	3+72	31249.76*	3199.103					
						14	6	31248.38	3199.244					
			3				14	31246.60	3199.426					A
			3′				3+46	31245.66*	3199.523					
			4				18	31226.19	3201.518	3201.512	−1	−0.006	OH	P
			4′				3	31225.30	3201.609					
						1, 15	2+5	31216.12	3202.551					
					16		5	31211.52	3203.023					
	2						8	31203.85	3203.810					
			5				21	31203.34	3203.863	3203.832	1	−0.031	Ti I	M
			5′				2+124	31202.23*	3203.977					
						16	4	31178.77	3206.388					
			6				22	31177.50	3206.518	3206.533	−1	+0.015	OH	P
			6′				2	31176.21	3206.651					
					17		4	31167.76	3207.521					
				2, 2′			6+2	31158.54	3208.469					
				3′			2	31157.54	3208.572					
				3			10	31156.80	3208.648					A
	3			1, 1′			10+3+2	31155.34	3208.799	3208.794	−2	−0.005	OH	P
				4′			2	31150.10	3209.338					
				4			14+35	31149.18*	3209.433	3209.434	−1	+0.001	OH	M
			7				24	31148.53	3209.500	3209.489	−2	−0.011	OH	P
			7′				2	31147.03	3209.655					
				5′			2	31137.29	3210.659					
				5		17	17+4	31136.04	3210.788					A
		1′					3+3	31124.70*	3211.958					
				6′			2	31119.28	3212.517					
					18		3	31118.57	3212.590					
				6			19	31117.96	3212.653					A
			8				24	31116.24	3212.831					A
			8′				1	31114.57	3213.003					
	4						11	31104.99	3213.993					A
		2, 2′					3+4	31097.53	3214.764					
				7′			1	31096.72	3214.848					
				7			20	31095.16	3215.009	3215.029	−2*N*	+0.020	OH	P
						18	3	31087.49	3215.803					
			9				24	31080.44	3216.532	3216.546	0	+0.014	Cr II	M
			9′				1	31078.50	3216.732					
				8′			1	31069.52	3217.662					
				8			21	31067.82	3217.839	3217.841	1	+0.002	Ni I	M
		3, 3′					5+4+58	31065.67*	3218.061					
					19		3	31063.77	3218.258					
	5						13	31052.42	3219.435	3219.429	−3	−0.006	OH?	P
			10				22	31041.05	3220.613	3220.607	−1	−0.006	Co I	M
			10′				1	31038.92	3220.834					
				9′			1	31037.97	3220.933					
				9			21	31036.09	3221.130	3221.135	−2	+0.005	OH–Ti I	B
						19	2	31033.49	3221.398					
		4′					3	31028.02	3221.966					
		4					7	31027.31	3222.040					
					20		2	31103.28	3224.537					
				10′			1	31002.17	3224.653					
				10			20	30999.98	3224.881	3224.925	0	+0.044	Fe I	M
			11				21	30997.79	3225.093	3225.122	−3	+0.029	OH	P
	6						14	30997.24	3225.166					A
			11′				1	30995.51	3225.346					
		5, 5′					9+3+50	30984.97*	3226.443	3226.446	−1	+0.003	OH	M
						20	2	30973.74	3227.613					
				11			19	30959.61	3228.086	3228.103	1	+0.017	Mn I	M
			12				19	30950.83	3230.002	3229.990	1	−0.012	Fe I	M
	7						14	30939.35	3231.201	3231.222	−3	+0.021	OH?–Ce II	B
		6′					3	30937.73	3231.370					
4		6					1+10+17	30936.75*	3231.473	3231.472	−1	−0.001	OH	P
				12			18	30914.94	3233.752	3233.762	−1	+0.010	V II	M
			13				17	30899.79	3235.338	3235.327	0	−0.011	Fe IP	M
		7′					2	30885.95	3236.787					
		7					11	30884.62	3236.926	3236.923	−2	−0.003	OH	P
	8						14	30878.47	3237.571	3237.583	−2	+0.012	OH	P
				13			16	30865.95	3238.885	3238.897	−1*N*	+0.012	OH–	B
5							1+3	30863.24*	3239.170					
			14				15	30844.71	3241.115	3241.138	−3	+0.023	OH?–Sm II	B
		8′					2	30830.03	3242.658					
		8					12	30828.51	3242.819	3242.834	−2	+0.015	OH	P
	9						14	30814.55	3244.287					A
				14			14	30812.58	3244.495	3244.498	−1	+0.003	OH–	B
6			15				1+13	30785.44	3247.355					A
		9′					2	30770.13	3248.971					
		9					12	30768.41	3249.153					A
				15			12+9	30754.71*	3250.600	3250.637	3*N*	+0.037	Fe I	M
	10						13	30747.49	3251.364	3251.353	1*N*	−0.011	Fe IIP Sc II?	M
			16				11	30721.82	3254.080	3254.060	1*N*	−0.020	Mn I	M
		10′					1	30706.41	3255.713					
		10					12+1	30704.48*	3255.918	3255.901	6	−0.017	Fe II	M
7							1+1	30703.60*	3256.010					
				16			10+1	30692.47*	3257.192					A
	11						13	30677.20	3258.814	3258.783	3	−0.031	Fe II	M
			17				9+2+2	30653.79*	3261.302					A
		11′					1+2	30638.59*	3262.920					
		11					11	30636.79	3263.112	3263.133	−3	+0.021	OH	P
				17			9	30625.62	3264.302					
	12						12+1	30603.61*	3266.650	3266.676	3*N*	+0.026	Cr I–	M
			18				8	30581.21	3269.043					
		12′					1+1	30567.65*	3270.493					
		12					10	30565.36	3270.738	3270.749	−3*N*	+0.011	OH	P
				18			8	30553.98	3271.956					
	13						10+1	30526.69*	3274.881	3274.909	1	+0.028	Ni II	M
			19				7+17	30503.90*	3277.328					
		13					9	30490.25	3278.795					
				19			7	30477.58	3280.158					
	14						9	30446.32	3283.526					
			20				6	30421.81	3286.172					
		14					8	30411.45	3287.291					
				20			6	30396.26	3288.934					
	15						8	30362.54	3292.587					
			21				5	30334.62	3295.617					
		15					7	30328.91	3296.238					
				21			5	30309.73	3298.325					
	16						7	30275.12	3302.095					
		16	22				6+4	30242.63	3305.643	3305.627	−2*N*	−0.016	Fe II	M
				22			4	30217.95	3308.342					
	17						6	30184.11	3312.051					
		17					5	30152.65	3315.507					
			23				3	30144.34	3316.421					
				23			3+3	30120.44*	3319.053					
	18						5	30089.32	3322.485					
		18					4+3	30058.68*	3325.873					
	19						4	29990.76	3333.405					
		19					4	29960.78	3336.741					
	20						3	29888.18	3344.846					
		20					3	29858.81	3348.136					
	21						3	29781.53	3356.825					
		21					2	29752.48	3360.072					
	22						2	29670.76	3369.367					
		22					2	29642.30	3372.592					

* Blend.

## Footnotes

1 Figures in brackets indicate the literature references on page 280.
